# Supplementary material for: Trends and spatial distribution of animal bites and vaccination status among victims and the animal population, Uganda: A veterinary surveillance system analysis, 2013–2017
Source: PLoS Negl Trop Dis. 2021 Apr 19;15(4):e0007944. doi: 10.1371/journal.pntd.0007944 (PMC8084341; doi:10.1371/journal.pntd.0007944)
Supplement: Attachment — Submitted filename: Responses to reviewer 3.docx [file pntd.0007944.s001.docx]

**Responses to reviewer 3**

| **Comments** | **Response** |
| --- | --- |
| Reviewer #3: My main concern regarding the present manuscript is in the methods section, which is poorly described and I missing a lot of information. The authors need to provide details on how the study was designed, and the statistical methods used. | I have added the more details in the methods |
| Reviewer #3: My main concerns in the results section is how the statistical analysis was performed. I do not know if the authors used appropriated statistical tests. | Yes, we used appropriate tests. For example, for trends analysis, we used logistic regression.  The rest of the computations were only descriptive |
| Line 58: All mammals? Is it not too much? | Revised to warm-blooded animals |
| Line 64: I recommend changing the word “Almost” for something like… | Changed the word “Almost” |
| Lines 65-73: I don’t think is necessary to describe the clinical course of the disease in dogs and humans. I recommend suggest deleting the entire paragraph. | Deleted |
| Line 75: “Prevention of rabies in animals is primarily achieved through vaccination”. This is the focus of your manuscript. | Yes |
| Lines 92-93: Please clarify why the cost of rabies vaccine is “prohibitive”. | The high cost of the human rabies vaccine makes some animal bite victims shy away from accessing rPEP because they cannot afford. |
| Lines 95-96: This first sentence looks confuse. Please rewrite to have a better flow, or I recommend deleting this sentence. | Rephrased it. |
| Lines 101-102: “However, Uganda also captures rabies-related data in a veterinary surveillance system, at National Animal Disease Diagnostic Epidemiology Centre (NADDEC)”. I think this sentence seems disconnected. However, Uganda also captures rabies-related data, and…? I recommend merging with the next sentence to have a better flow. | I have merged with the next sentence to have a better flow |
| Line 103: Please clarify “mandated”. | Deleted it |
| Major: The introduction section is too long and makes it lose the main focus of the manuscript. My suggestion is to delete some excessive information and focus on what your study brings of most important. | Deleted some information |
| Lines 117-118: “The officer visits the scene of the incident to ensure that the animal (usually dog/cat) is killed and its head packaged for shipment to NADDEC for analysis”. The office always kills the SUSPECTED animals? Is there any observation at the first moment? Please clarify. | In most cases, by the time the officer arrives, the community will have killed the suspected rabid dog or the dog could have escaped. |
| Lines 126-131: I recommend creating another topic to explain the variables were capture for further analysis, giving more details and adding information about inclusion and exclusion criteria. | Rephrased and added more information |
| Lines 139-141: Same here, please provide further details about using laboratory records. | Provided more details. |
| Lines 145-146: “Regional and national human population data were obtained from the Uganda National Census 2014” and? What other information did the authors collect? Please provide details. | Deleted |
| Lines 156-159: Great, but how did the authors calculate this? | 21 animal bites per 100,000 population (new animal bites divided by human population at risk), 8.2/100,000 in 2013 (new animal bites in the year divided by the population at risk in 2013), (OR=0.62, p=0.0074) (Trends analysis using logistic regression Epiinfo version 7.2.2.6) |
| Lines 171-174: What does it mean “6,997 pets were reported destroyed by the communities”? Please clarify. | According to rabies surveillance information we analysed from 2013-2017, 6997 suspected rabid pets were destroyed by the communities |
| Lines 187-192: I think authors should also include here a discussion about the increase of animal bites in 2014. | This is explained by the increase in reporting during that period |
| Lines 202-204: “Vaccinating pets…” Only pets? Please clarify. | Clarification: “Vaccinating pets is one of the most important ways to prevent rabies infections among both animals and humans”. Vaccinating pets is one of the most important. It does not mean vaccinating only pets. |
| Any recommendations regarding education of the population? | Its included |
