# Supplementary material for: Trends and spatial distribution of animal bites and vaccination status among victims and the animal population, Uganda: A veterinary surveillance system analysis, 2013–2017
Source: PLoS Negl Trop Dis. 2021 Apr 19;15(4):e0007944. doi: 10.1371/journal.pntd.0007944 (PMC8084341; doi:10.1371/journal.pntd.0007944)
Supplement: Attachment — Submitted filename: Responses to reviewer 2_V19Oct2020.docx [file pntd.0007944.s002.docx]

| **Comments to reviewer 2** | **Responses to reviewer 2** |
| --- | --- |
| Methods:  Reviewer #2: The objectives of the study are clear and the methods used are standard for this type of analysis. The manuscript is evaluating a public health surveillance system and no ethical or regulatory concerns are apparent. | None |
| Results:  Reviewer #2: The results match the proposed analysis and have been clarified over the previous version. Figures are adequate for this type of information  What is the total population of Uganda that was used to calculate incidence?  How many records were removed for unknown district or date?  line 179: suggest authors replace 'received' with 'initiated' | None  33,491,707 was used as the total population of Uganda to calculate incidence.  2 records were removed  Replaced 'received' with 'initiated' |
| Conclusions:  Reviewer #2: The public health relevance of this topic is important and the authors provide some generalizations from their analysis that highlight the issues with rabies surveillance in Afria. They highlight how changing from active to passive surveillance can influence reporting rates and by extension incidence. While the limitations of the analysis are presented, I would caution that some of their conclusions about overall rabies risk in Uganda could be more nuanced. | None |
| **Editorial and Data Presentation Modifications?**  Reviewer #2: The current version clarifies many of the issues of the original version. Some minor editing of language and punctuation my improve the overall readability of the manuscript. | Improved on the language and punctuation |
| **Summary and General Comments**  Reviewer #2: The manuscript provides important information for persons interested in canine rabies elimination. It highlights many of the issues faced by government institutions in low resource settings including incomplete reporting, unreliable population data, laboratory testing capacity, and inability to follow patient outcomes | None |
