# Supplementary material for: Trends and spatial distribution of animal bites and vaccination status among victims and the animal population, Uganda: A veterinary surveillance system analysis, 2013–2017
Source: PLoS Negl Trop Dis. 2021 Apr 19;15(4):e0007944. doi: 10.1371/journal.pntd.0007944 (PMC8084341; doi:10.1371/journal.pntd.0007944)
Supplement: Attachment — Submitted filename: Response_Reviewer1.docx [file pntd.0007944.s003.docx]

| **Comment** | **Response** |
| --- | --- |
| Reviewer #1: Thank you to the authors for their thorough revisions in response to previous comments, which have improved the clarity of conclusions drawn from the results. All concerns in the text have been addressed, however some final adjustment to Figure 1 would considerably benefit the clarity of communicating key points in the data.  I'm sorry for poorly communicating the suggestion of a stacked graph in previous comments. As opposed to stacked bar graphs, I was trying to suggest stacked line graphs with each plotted on an independent y-axis. This avoids the need for additional labelling / colour which can hamper interpretation. Each axis has it's own scale and label as opposed to grouping multiple variables into one axis, allowing the reader to immediately understand what data is being shown in each line. An example of this approach can be seen here: http://www.performance-ideas.com/2012/03/27/stacked-line-charts/  Other than this concern the manuscript appears suitable for publication and would be of interest to the readership of PLOS NTD. | Drawn stacked line graphs as advised (See revised fig1) |

**Comments and Responses from reviewer 1**
